# Sardinian Type 1 diabetes patients, Transthyretin and *Mycobacterium avium subspecies paratuberculosis* infection

**DOI:** 10.1186/1757-4749-4-24

**Published:** 2012-12-27

**Authors:** Speranza Masala, Davide Cossu, Adolfo Pacifico, Paola Molicotti, Leonardo A Sechi

**Affiliations:** 1 Department of Biomedical Sciences, Division of Experimental and Clinical Microbiology, University of Sassari, Sassari, Italy; 2 Servizio di Diabetologia, Istituto di Clinica Medica, University of Sassari, Sassari, Italy

**Keywords:** *Mycobacterium avium* subsp. *paratuberculosis*, Type 1 diabetes, Transthyretin, Biomarker, Sardinia

## Abstract

**Background:**

*Mycobacterium avium* subsp. *paratuberculosis* (MAP) is the cause of Johne’s disease, an enteric granulomatous disease. Recently, MAP has been associated with different autoimmune diseases such as Crohn’s disease, type 1 diabetes (T1D) and multiple sclerosis. Transthyretin (TTR) is a plasma transport protein for thyroid hormone and forms a complex with retinol-binding protein. Reduced TTR plasma levels in MAP infected ovines have been reported.

TTR exerts also a functional role in the pancreas promoting insulin release and protecting β-cells from death.

Our objective was to identify a protein that could be used as a diagnostic marker of T1D for determining disease progression and monitoring at-risk patients. We postulate that serological TTR levels would be reduced in T1D MAP exposed patients. Our hypothesis is based on the observation of cases of T1D patients with decreased TTR levels beside the reduced TTR plasma levels in ovines with Johne’s disease.

We quantified the plasma protein levels of TTR in 50 people with T1D and 51 age-matched healthy controls (HCs) by means of enzyme-linked immunosorbent assays (ELISA).

**Findings:**

Our pilot study showed that plasma TTR levels were not significantly lower/higher in T1D Sardinian cases compared to the HCs.

**Conclusion:**

These preliminary data indicate that plasma TTR may not be a good candidate biomarker for T1D diagnosis and further studies to elucidate the possible link are needed.

## Background

T1D is a chronic autoimmune disease characterized by progressive T cell mediated β-cell destruction. The presence of autoantibodies (aAbs) against islet antigens is a hallmark for the development of T1D [1]. Islet aAbs titers against glutamic acid decarboxylase (GAD65), insulinoma associated protein-2 (IA-2), and zinc transporter 8 (Znt8) are routinely measure out alone or in combination as biomarkers for T1D risk assessment. They are as well useful in the identification and characterization of adult-onset autoimmune diabetes [2]. Notwithstanding the aforementioned biomarkers, the existing tests for T1D prediction are still imperfect and newer biomarkers need to be found. Transthyretin (TTR) is a 55 kDa homotetramer and it is the major carrier of serum thyroxine, tri-iodothyronine, and vitamin A (retinol) through association with retinol-binding protein [3].

TTR exerts also a functional role in the pancreas promoting insulin release and protecting β-cell from death [4]. Interestingly a mass spectrometry surface-enhanced laser desorption ionisation time of flight (SELDI TOF)-based proteomics study has recently been used to identify potential ovine paratuberculosis diagnostic markers. Analysis of the whole serum led to the identification of TTR as putative biomarker in sheep with MAP exposure [5]. Moreover another protein-based study using the isobaric tag for relative and absolute quantitation (iTRAQ) method showed that TTR is significantly increased in paratuberculosis infected cattle compared to controls [6]. In addition, it was reported that TTR plasma concentration is decreased in human subjects with T1D [7].

We wanted to explore if TTR would be a suitable biomarker for T1D risk assessment in *Mycobacterium avium* subspecies *paratuberculosis* (MAP) exposed individuals.

We have previously demonstrated that MAP infection could be a risk factor for T1D in the Sardinian population [8]. To assess TTR eligibility as T1D diagnostic markers, we investigated whether TTR serum levels would be reduced in Sardinian T1D patients compared to HCs. Serum samples of 50 T1D and 51 HCs were analyzed by MBS Human-TTR ELISA kit.

## Methods

### Sera

The sera of T1D patients (n = 50; mean age at onset 33.5 ± 7 years), diagnosed according to the American Association of Diabetes criteria [9] and HCs (n = 51) age-matched with T1D patients (mean age 36.36 ± 7 years) were obtained from the University Hospital of Sassari in agreement with the local ethical committee guidelines. All the samples were collected from patients and individuals who did not have any concurrent chronic or autoimmune diseases. All the collected sera were stored in −20°C until measurements were carried out. Written informed consent to take part in the study was obtained from all subjects according to the guidelines of the Institutional Ethical Committee.

### ELISA

ELISA was carried out, as described elsewhere [8] to detect Abs specific for MAP3865c peptides.

The concentration of TTR in serum samples from T1D patients and matched HCs was determined using an ELISA kit (MBS, The Netherlands) following the manufacturer’s instructions.

### Statistical evaluation

The statistical significance of the data was determined by both the Fisher’s exact test and the independent Student’s t-test using Graphpad Prism 5.0 software. The p-value was calculated for each experiment carried out in triplicate.

## Result

Ab positivity against MAP3865c peptides was detected in 45% of T1D and only in 8.5% of HCs (Fisher’s exact test; p < 0.0001), showing a statistically significant higher frequency in T1D.

**Figure 1:**
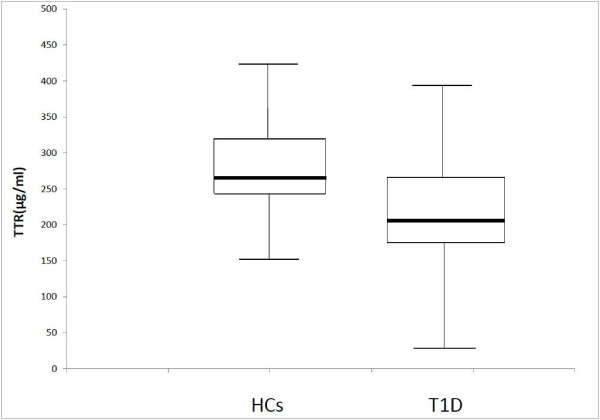
**Acquired TTR ELISA data are displayed by a box plot.** The calculated median concentration is 285 μg/ml in HCs and 207.6 μg/ml in T1D patients.

TTR serum concentrations on 50 T1D and 51 HCs subjects were assessed performing ELISA according to the (kit) manufacturer’s instructions. The determined median concentrations of TTR in the sera from HCs were 285 μg/ml and 207.6 μg/ml for T1D patients. In Figure 1 data are shown in a box plot. The HCs group showed a mean of 289.5 μg/ml TTR protein and the T1D group a mean of 217.5 μg/ml (p < 0.0001). When dividing TTR serum levels according to MAP3865cAb positivity, the MAP3865cAb + T1D patients showed a lower TTR concentration compared to the HCs (184.2 μg/ml vs 347.6 μg/ml; p < 0.0001) (Table 1). However there was no correlation between positivity for anti-MAP3865c Abs and lower/higher TTR serum level, as in both cases data fit in the physiological range. In fact, according to UniProt Declaration, the normal fluctuation of TTR in the sera should range from 100 μg/ml to 400 μg/ml. Unfortunately, insulin and glucose levels were not available for all the patients. Up until now a relationship between reduced TTR levels and T1D in a MAP infected population could not be demonstrated by the use of ELISA even if a trend towards TTR down-regulation in T1D MAP3865cAb + patient was identified (184.2 μg/ml vs 347.6 μg/ml p < 0.0001).

**Table 1:** Plasma transthyretin (TTR ) concentration (μg/ml) determined by ELISA

	**T1D**	**HCs**	**p values**
TOTAL	217.5 ± 69.8	289.5 ± 63	p < 0.0001
MAP3865c Ab +	184.2 ± 59	347.6 ±34	p < 0.0001
MAP3865c Ab -	248.3 ± 64.3	284.5 ± 61.9	p = 0.0216

*Values* (μg/ml) *are MEAN ± SEM.*

Levels of TTR protein (mean ± SE) are reported for control subjects (HCs) and type 1 diabetes (T1D) patients. Furthermore, T1D and HCs mean were as well calculated dividing for MAP3865cAb + and MAP3865cAb-.

## Conclusion

Blood-based biomarkers have an advantage in that they are suitable for large scale studies with the ease of venepuncture and allowing for repeatability in clinical settings.

A previous proteomics study using SELDI-TOF showed decreased levels of TTR in plasma of MAP exposed sheep [5]. Conversely an increase in plasma TTR levels was found in MAP infected cattle [6]. Noteworthy low levels of vitamin A, correlating with reduced TTR and raised concentrations of C-reactive protein, have been reported in patients with tuberculosis [10,11].

Given some studies linking MAP to T1D [8], and the reported reduction of TTR plasma levels, in T1D patients compared to HCs [7], we sought to investigate if MAP infection is associated to TTR down-regulation in humans T1D subject.

We consider a new approach for diagnosing T1D using serum from patients with the disease and HCs all from Sardinian region. In this preliminary study we were interested to analyze TTR profiles as diagnostic markers and to infer if TTR distinguished T1D from HCs.

We were not able to find TTR levels below threshold in T1D and MAP3865cAb + patients.

Our findings, indeed, showed that there is not a reduction of TTR serological level in Sardinian T1D MAP exposed individuals, but if we divide the population under scrutiny according to MAP3865cAb+, we find out that T1D group display diminished TTR (mean) concentration. It is as well important to check TTR potential to be a prognostic marker of T1D, and to accomplish this goal it is mandatory to set up a follow-up study, to understand if the lack of correlation could be explained taking into account the disease stage. Is it the same in recent onset patients or at 6 months, 1 year or 2 years after the onset?

However, a drawback of this study could the limited number of subjects. A larger study population and different patient cohorts (not Sardinian) as well should be assessed to fully answer the question if T1D patients and at risk subjects could be identified on the basis of a significantly lower TTR serum concentration.

## Abbreviations

TTR: Transthyretin; aAbs: auto-antibodies; T1D: Type 1 diabetes; HCs: Healty controls; ELISA: Enzyme-linked immunosorbent assays; GAD65: Glutamic acid decarboxylase; IA-2: Insulinoma associated protein-2; Znt8: Zinc transporter 8; SELDI TOF: Surface-enhanced laser desorption ionisation time of flight; MAP: *Mycobacterium avium* subspecies *paratuberculosis*; iTRAQ: Isobaric tag for relative and absolute quantitation.

## Competing interests

The authors declare that they have no competing interests.

## Authors’ contributions

MS: carried out the experiments, the statistical analysis and wrote the manuscript; DC: have been involved in drafting the manuscript; AP: Contributed providing samples; PM helped in performing data analysis; LAS conceived, coordinated the study and wrote the manuscript. All authors read and approved the final manuscript.

## Authors’ information

SM, DC, PM, LAS: Department of Biomedical Sciences, Division of Experimental and Clinical Microbiology, University of Sassari, Sassari, ITALY. AP: Servizio di Diabetologia, Istituto di Clinica Medica, University of Sassari, Sassari, ITALY.
